# Mass spectrometry-based microbiological testing for blood stream infection

**DOI:** 10.1186/s12014-020-09278-7

**Published:** 2020-05-13

**Authors:** Fumio Nomura, Sachio Tsuchida, Syota Murata, Mamoru Satoh, Kazuyuki Matsushita

**Affiliations:** 1 Division of Clinical Mass Spectrometry, Chiba University Hospital, 1-8-1 Inohana, Chuo-ku, Chiba, 260-8677 Japan; 2 Division of Laboratory Medicine, Chiba University Hospital, 1-8-1 Inohana, Chuo-ku, Chiba, 260-8677 Japan

**Keywords:** Matrix-assisted laser desorption ionization–time of flight mass spectrometry (MALDI-TOF MS), Liquid chromatography (LC) coupled with tandem mass spectrometry (MS/MS), Blood stream infection, Bacteremia, MALDI Biotyper, Vitex MS, Sepsityper, Rapid BACpro II, Centrifugation and membrane filtration technique (CMFT)

## Abstract

**Background:**

The most successful application of mass spectrometry (MS) in laboratory medicine is identification (ID) of microorganisms using matrix-assisted laser desorption ionization–time of flight mass spectrometry (MALDI-TOF MS) in blood stream infection. We describe MALDI-TOF MS-based bacterial ID with particular emphasis on the methods so far developed to directly identify microorganisms from positive blood culture bottles with MALDI-TOF MS including our own protocols. We touch upon the increasing roles of Liquid chromatography (LC) coupled with tandem mass spectrometry (MS/MS) as well.

**Main body:**

Because blood culture bottles contain a variety of nonbacterial proteins that may interfere with analysis and interpretation, appropriate pretreatments are prerequisites for successful ID. Pretreatments include purification of bacterial pellets and short-term subcultures to form microcolonies prior to MALDI-TOF MS analysis. Three commercial protocols are currently available: the Sepsityper^®^ kit (Bruker Daltonics), the Vitek MS blood culture kit (bioMerieux, Inc.), and the rapid BACpro^®^ II kit (Nittobo Medical Co., Tokyo). Because these commercially available kits are costly and bacterial ID rates using these kits are not satisfactory, particularly for Gram-positive bacteria, various home-brew protocols have been developed: 1. Stepwise differential sedimentation of blood cells and microorganisms, 2. Combination of centrifugation and lysis procedures, 3. Lysis-vacuum filtration, and 4. Centrifugation and membrane filtration technique (CMFT). We prospectively evaluated the performance of this CMFT protocol compared with that of Sepsityper^®^ using 170 monomicrobial positive blood cultures. Although preliminary, the performance of the CMFT was significantly better than that of Sepsityper^®^, particularly for Gram-positive isolates. MALDI-TOF MS-based testing of polymicrobial blood specimens, however, is still challenging. Also, its contribution to assessment of susceptibility and resistance to antibiotics is still limited. For this purpose, liquid chromatography (LC) coupled with tandem mass spectrometry (MS/MS) should be more useful because this approach can identify as many as several thousand peptide sequences.

**Conclusion:**

MALDI-TOF MS is now an essential tool for rapid bacterial ID of pathogens that cause blood stream infection. For the purpose of assessment of susceptibility and resistance to antibiotics of the pathogens, the roles of liquid chromatography (LC) coupled with tandem mass spectrometry (MS/MS) will increase in the future.

## Background

Mass spectrometry (MS) is a powerful analytical tool that measures the mass to charge ratio (*m/z*) of one or more molecules in a sample. Compared with conventional methods such as immunoassays, MS can detect multiple analytical targets of interest simultaneously with improved specificity. MS technology has become significantly robust and sophisticated, leading to increasing adoption of MS in various subdisciplines of laboratory medicine (Table 1). Liquid chromatography (LC) coupled with tandem mass spectrometry (MS/MS) is widely used in clinical medicine; it has been used for newborn screening [1], toxicology [2], therapeutic drug monitoring [3], endocrinology [4], and more recently for targeted proteomics [5]. Another novel application of matrix-assisted laser desorption ionization (MALDI)-MS is imaging MS [6]. MALDI imaging has enabled label-free, multiplex measurement of a wide variety of molecules in tissue samples together with their spatial localization. Indeed, rapid MALDI-MS imaging of surgical tissue specimens has been proposed for intraoperative quick diagnostics [7].

**Table 1:** Applications of mass spectrometry to laboratory medicine

GC/MS
Toxicology
Inborn errors of metabolism
Metabolomics
MALDI-TOF MS
Clinical microbiology
Imaging MS
LC/MS/MS
Inborn errors of metabolism
Therapeutic drug monitoring
Toxicology
Endocrinology
Targeted metabolomics, peptidomics and proteomics
Clinical microbiology

The most successful application of MS in laboratory medicine is identification (ID) of microorganisms using MALDI–time of flight MS (MALDI-TOF MS).

Identification of bacteria using MALDI-TOF MS was initially applied to bacterial colonies grown on agar plates. Because the method is simple, easy to perform, and rapid, it has been increasingly used in clinical microbiology laboratories; indeed, a revolutionary shift in clinical diagnostic microbiology has occurred all over the world [8–13].

Direct analysis of clinical specimens without the need for prior colony formation increases the usefulness of this technology. Urine is a target for this purpose [14, 15]. Cerebrospinal fluid is another good target; we previously reported that MALDI-TOF MS can provide rapid ID of bacteria in cerebrospinal fluid, thus enabling early and appropriate treatment [16]. From the practical point of view, the most promising clinical specimen for direct testing is positive blood cultures; rapid and accurate ID of causative microorganisms is essential for early initiation of appropriate antimicrobial therapy [17, 18]. Because blood culture bottles contain a variety of nonbacterial proteins that may interfere with the analysis and interpretation of bacterial proteome profiles, pretreatment to effectively remove host proteins and blood cells while also concentrating the microorganisms is a key step for successful ID [19].

In this review, we describe MALDI-TOF MS-based bacterial ID with particular emphasis on the methods so far developed to directly identify microorganisms from positive blood culture bottles with MALDI-TOF MS including our own protocols. We touch upon the increasing roles of LC/MS/MS as well.

## History of bacterial ID using MS

MS analysis of volatile pyrolysis products to identify complex organic materials was reported in 1952 [20]. In the 1960s, technical progress was made due to the combination of pyrolysis with gas–liquid chromatography (Py-GLC); the values of Py-GLC for the classification and ID of bacteria and other microorganisms were demonstrated [21, 22]. Furthermore, reproducible fingerprinting of bacteria using the Curie-point pyrolyzer and quadrupole mass spectrometer combination was reported [23]. A well-recognized report by Anhalt and Fenselau entitled “Identification of bacteria using mass spectrometry” was published in 1975 [24]. The hard ionizations used in these initial reports allowed detection of mainly bacterial lipids, resulting in limited differentiation of bacteria at the species level. The soft ionization technique [25] has allowed differentiation and classification of microorganisms based on their proteins and peptides.

Differentiation of bacteria based on their protein profiles from MALDI-TOF MS after release of cell contents by breaking cell membranes with sonication was reported in 1994 [26]. In 1996, two reports indicated that MALDI-TOF mass spectral “fingerprints” could be simply and rapidly obtained from whole bacterial cells without any pretreatment before the MS analysis [27, 28], opening the door to simple and rapid MS-based bacterial ID. However, more than 10 years elapsed until the first report indicated that MALDI-TOF MS–based ID was suitable for routine use in clinical microbiology laboratories. The possible reasons for this delay were summarized in a review by Welker [29] as follows:

1. The general view that the proteome is very dynamic in living cells, and hence, the pattern of protein expression would be subject to changes in response to growth conditions;
2. Doubts as to whether differences and similarities in mass spectral patterns are reliably consistent with the established taxonomy;
3. A lack of comprehensive databases covering all clinically relevant species;
4. Compared with conventional methods, the MS-based ID procedures seemed too simple compared with the complexity of the task;
5. A method that would enable an untrained person to identify a microorganism in only a fraction of the time required by an expert using the conventional methods seemed unreliable;
6. Most of the early publications involving MALDI-TOF MS–based ID appeared in journals most microbiologists do not often access.

## MALDI-TOF MS for bacterial ID from culture plates

ID is made by matching ionized proteins and peptide peak profiles that are unique to the particular microorganisms to established spectral databases.The final mass spectral signature is composed of peaks ranging from 1,000 to 30,000 *m/z*, and peaks between 2 and 20 kDa are mostly used because they are stable and have a strong signal-to-noise ratio. In an effort to understand why some proteins are detectable while many others are not, Ryzhov and Fenselau characterized various features of the proteins that are detectable in MALDI-TOF MS analyses of whole *Escherichia coli* cells; 40% of them were ribosomal proteins, followed by DNA-binding proteins and cold-shock proteins [30].

For MALDI-TOF MS–based bacterial ID in clinical microbiology laboratories, two systems (including the associated databases) are widely used: the MALDI Biotyper (Bruker Daltonics; http://www.bruker.com/jp/products/mass-spectrometry-and-separations/literature/literature-room.html?eID=dam_frontend_push&stream=1&docID=58883) and the VITEK MS (bioMerieux; http://www.biomerieux-diagnostics.com/sites/clinic/files/9300819-002-gb-a_vitek-ms.pdf). As of September 2019, approximately 275 instruments (3/4 of them are Bruker’s MALDI Biotyper, and 1/4 bioMérieux’s VITEK^®^MS) were in operation for routine use in clinical microbiology labs in Japan (Personal communication).

Although the analytical principles of the two systems are similar, differences are present in the way the databases and the diagnostic algorithms are constructed. Also, the ways to present the ID results are different.

In the MALDI Biotyper system, the results of the pattern-matching process are expressed as proposed by the manufacturer with scores ranging from 0 to 3; scores below 1.7 are regarded as an unreliable ID; scores between 1.7 and 2.0 as genus-level ID; and scores > 2.0 are regarded as species level. The Vitex MS system generates a confidence score indicated as the percent probability. The range of percent probabilities for a correct ID is from 60 to 99 with values closer to 99.9 indicating a closer match. When the obtained percent probability is under 60, it is considered a non-ID. For both systems, however, lowering the cut-off values for bacterial ID from blood culture bottles increases the sensitivity of the test without compromising the specificity [31, 32].

Diagnostic values of bacterial ID from culture plates using MALDI-TOF MS are well established: for Gram-negative bacteria, 92.5–99.8% at the genus level and 91.7–98.2% at the species level; for Gram-positive bacteria, 92.5–95.5% and 91.7–92.8%, respectively [13]. This technology is increasingly used for ID of mycobacteria [33], molds [34], and also Nocardia species [35].

## ID from monomicrobial positive blood cultures with MALDI-TOF MS

**Fig. 1:**
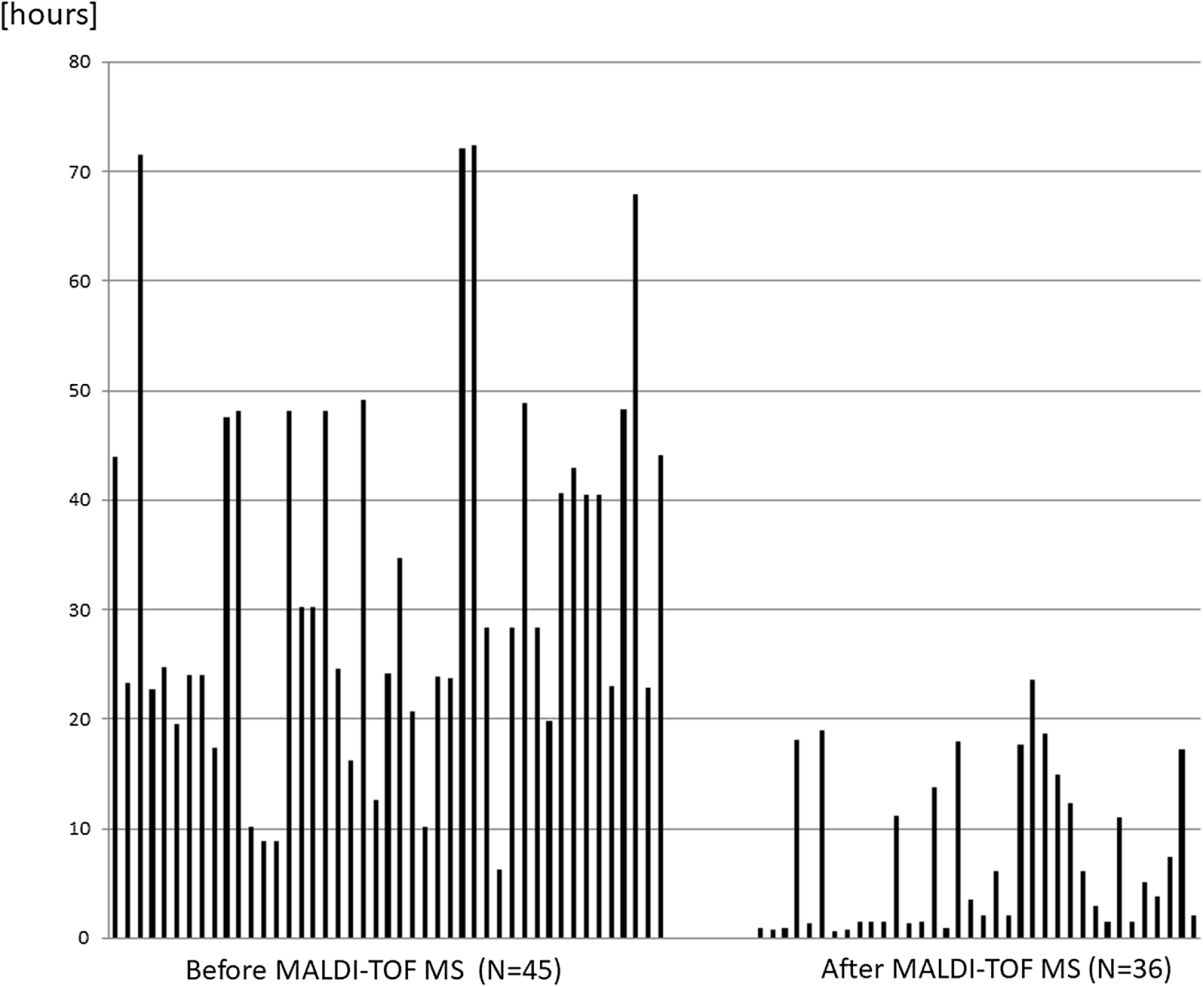
The time required from the point when blood culture bottles are flagged as positive to reporting of the pathogenic bacterial species before and after MALDI-TOF MS implementation in the clinical microbiology laboratory at Chiba University Hospital

Direct ID of bacteria from blood culture bottles is a promising application of MALDI-TOF MS. The conventional way to identify microorganisms responsible for blood stream infection involves an overnight subculture from a positive blood culture followed by species-level ID and antimicrobial susceptibility testing with an automated system, which takes 18–24 h, whereas MALDI-TOF MS can provide ID results in less than 1 h using purified bacterial pellets obtained from blood culture bottles. The reduction in time required for blood culture bottles flagged as positive for reporting of the bacterial species before and after MALDI-TOF MS use in our hospital is shown in Fig. 1.

The averages of lengths of time before and after introduction of MALDI-TOF MS were 33 h and 7 h, respectively. Even after introduction of MALDI-TOF MS, significant lag time was noted in several samples; in some cases, it was due to availability of staff to perform the analysis once a culture bottle was flagged as positive. In the other cases, direct ID from positive culture bottle was not successful, particularly for *Staphylococcus epidermidis*, and ID was made possible only after colony was formed in the solid subculture media.

As blood culture bottle media contain a variety of nonbacterial proteins—including those derived from blood cells—that may interfere with the interpretation of bacterial proteome profiles, pretreatment to substantially remove these host proteins is necessary for accurate ID of causative microorganisms. Basically, pretreatment can be done in two ways: purifications of bacterial pellets and short-term subcultures (microcolonies).

A number of studies have reviewed the performances of MALDI-TOF MS-based rapid microorganism ID from positive blood cultures [19, 32]. The overall performance results of these studies vary greatly, ranging from 60 to 99% ID rates at the species level. Direct comparison of these studies is difficult because the ID rates depend on the pretreatment method, the volume of the blood sample, the distribution of the microbial isolates, and the definition of the cut-off levels for species-level ID.

### I Purification of bacterial pellets

1. Commercially available kits
  With increasing regulatory demands for quality assurance of in vitro diagnostic products, maintenance of the so-called “laboratory-developed test” is challenging for individual clinical microbiology laboratories. For this reason, clinical laboratories prefer to use commercially available diagnostic products, for which regulatory approval is provided by the manufacturer. To our knowledge, three commercial protocols are available at present: the Sepsityper^®^ kit (Bruker Daltonics) [36], the Vitek MS blood culture kit (bioMerieux, Inc.) [37], and the rapid BACpro^®^ II kit (Nittobo Medical Co., Tokyo, Japan) [38].
  i. The Sepsityper^®^ kit (https://www.bruker.com/products/mass-spectrometry-and-separations/ivd-ce-certified-maldi-biotyper/mbt-sepsityper-ivd-kit.html). The Sepsityper^®^ kit is the most widely used commercial kit. Indeed, the MALDI Sepsityper^®^ kit was used as a representative pretreatment protocol for the MALDI-TOF MS-based method for the report of a parallel evaluation of MALDI-TOF MS- and microarray-based tests for the rapid ID of Gram-negative bacilli from positive blood cultures [39]. The MALDI Sepsityper^®^ kit involves the lysis of blood cells, followed by centrifugation and washing steps. Briefly, an aliquot of positive blood culture medium is mixed with 200 µl of the Sepsityper^®^ lysis reagent. The mixture is centrifuged, and the supernatant is decanted; the pellets are subjected to washing with serial centrifugation steps, and the final pellets are applied to a target plate directly or after extraction steps. Since the initial report [36], a growing number of reports using this kit has been published as reviewed by Morgenthaler and Kostrzewa [40]. According to a meta-analysis of data from 21 reports, the Sepsityper kit allowed ID at the species level of 80% of 3,320 positive blood culture bottles. Gram-negative bacteria were identified at a higher rate (90%) than Gram-positive bacteria (76%) and yeast (66%); no apparent misidentifications at the genus level were reported at a cut-off of 1.6.
  ii. The Vitek MS blood culture kit (https://www.biomerieux-diagnostics.com/vitekr-ms-accessories-reagents). The Vitek MS blood culture kit, initially described by Fothergill et al. [37], is a filtration-based method. Briefly, an aliquot of positive blood culture medium is incubated with lysis buffer, and the lysate is vacuum-filtered through a membrane with a manifold. The membrane is washed, and the bacterial film remaining on the filter is scraped off and placed on a target plate. Processing with the Vitek MS blood culture kit resulted in ID rates similar to those of the MALDI Sepsityper. [37, 41]. The Vitek kit is unique because no centrifugation steps are involved.
  iii. The rapid BACpro^®^ (https://nittobo-nmd.co.jp/english/special/rapidBACpro.html)The rapid BACpro^®^ II kit (Nittobo Medical Co., Tokyo, Japan) is the most recently released commercial pretreatment protocol using cationic particles. The prototype was reported by Ashizawa and Murata [38]. In this protocol, a polyallylamine-polystyrene copolymer is used to collect microorganisms in positive blood culture bottles. Because the majority of bacteria are adherent to positively charged polymer surfaces, the copolymer has been used in wastewater treatment [42]. Bacterial aggregation via copolymerization of polyallylamine and polystyrene results in macroscopically visible aggregates [38]. Briefly, in this protocol, blood cells are separated from the culture broth with centrifugation. The resulting pellets on the surface of the separating gel are collected with suspension buffer and mixed with copolymer solution and magnesium chloride solution, followed by three centrifugation steps. The second pellet is treated with 30 μl of 70% formic acid and 30 μl of acetonitrile, centrifuged, and a portion of the final supernatant is placed on a MALDI plate. The advantages of this novel protocol over the conventional ways are the relatively short runtime (approximately 15 min) and that the entire procedure can be completed within a biosafety cabinet using a simple benchtop centrifuge.
    This original protocol, initially intended for use with the MALDI Biotyper, was modified and improved by Watari and coworkers [43] for use with the Vitek MS system. This improved protocol can be applied to the MALDI Biotype system as well.
    We evaluated the performance of the rapid BACpro^®^ II kit compared with the Sepsityper^®^ Kit using 113 monomicrobial positive blood cultures [44]. Although preliminary, the overall MALDI-TOF MS-based ID rates were greater with the rapid BACpro^®^ II than with the Sepsityper^®^, with a score > 1.7 and > 2.0 obtained using the rapid BACpro^®^ II kit at 99.1% and 80.5%, respectively; for the Sepsityper^®^ kit, the percentages were 83.2% and 58.4%, respectively. The superiority of the rapid BACpro^®^ II was confirmed by Kaylin et al. [45], who performed a three-way comparison of the two commercial kits and their home-brew method.
2. Laboratory-developed tests for bacterial collection and purification
  Commercially available kits are useful, but they are costly, and bacterial ID rates using these kits are still not satisfactory, particularly for Gram-positive bacteria. In an attempt to reduce costs and hands-on time and also to increase the sensitivity of pathogen ID, a number of microbiology laboratories have developed their own pretreatment protocols. The following are representative examples of laboratory-developed protocols:
  (i) Stepwise differential sedimentation of blood cells and microorganisms [46]
    A portion of the positive blood culture is centrifuged at low speed to remove blood cells. The resultant supernatant is centrifuged at high speed to collect bacteria, which are subjected to washing and extraction, followed by MALDI-TOF MS analysis.
  (ii) Combination of centrifugation and lysis procedures to remove blood cells and collect bacteria Briefly, a portion of the positive blood culture is mixed with lysis buffer and centrifuged. Collected bacterial pellets are subjected to washing, extraction, and MALDI-TOF MS. In some studies [47, 48], blood collection tubes with separator gels were used to separate blood cells at the bottom and bacteria at a gel surface after centrifugation of the positive blood culture. Various reagents including saponin [49], ammonium chloride [50], triton [51], and sterile water [47] are used for the lysis step. The workflow of the Sepsityper^®^ kit belongs to this group.
  (iii) Lysis-vacuum filtration This protocol is similar to (ii), but relies on vacuum filtration instead of centrifugation to collect bacteria. This approach was originally described for the Vitek MS blood culture kit [37]. Because the Vitek MS blood culture kit is not readily accessible for non-Vitek MS users in Japan, we described an in-house lysis filtration method for use with the MALDI-Biotyper system [52].
    Our in-house method is similar to the original lysis filtration method [37], but differs from the previous technique in four ways. First, we used ammonium chloride for lysis of blood cells, as recommended by Prod’hom et al. [50]. Second, we added a 3-min centrifugation step to ensure bacterial recovery after filtration and washing of the membrane. Third, we used a 10-ml syringe rather than a vacuum pump for the filtration procedure. Fourth, we used 70% formic acid for on-plate extraction of bacterial proteins. Performance of the in-house protocol was evaluated using 117 monomicrobial positive blood cultures. Although preliminary, the overall MALDI-TOF MS-based ID rates with a score > 1.7 and > 2.0 obtained using the in-house protocol were 99.2% and 85.5%, respectively.
  (iv) CMFT This is a new in-house protocol in which vacuum filtration is coupled with differential centrifugation. We recently reported the details of the workflow and the preliminary results [53]. Briefly, a portion of positive blood cultures is initially subjected to Gram staining. A 3-ml sample of blood culture fluid is mixed with 3 ml of phosphate-buffered saline, vortexed, and centrifuged at 213×*g*; the appropriate centrifugation time is 3 min for Gram-negative organisms and 1 min for Gram-positive ones. The supernatant is collected and manually drawn into a 10-ml syringe fitted with a 0.45-µm membrane filter. Subsequently, each membrane is washed and placed into a 50-ml tube containing 1 ml distilled water, vortexed, and the resulting liquid is transferred to another centrifugation tube, which is centrifuged at 20,600×*g* for 3 min. The pellet formed at the bottom of the tube is recovered and applied to a MALDI target plate. CMFT requires only approximately 15 min to complete the whole workflow, compared with approximately 30 min for the Sepsityper^®^ protocol.
    We prospectively evaluated the performance of this novel method compared with that of the Sepsityper^®^ using 170 monomicrobial positive blood cultures. For Gram-negative bacterial isolates, the species-level ID rates obtained with the CMFT and the Sepsityper^®^ were comparable (98.8% vs. 92.9%, respectively). In contrast, for Gram-positive isolates, the performance of the CMFT was significantly better than that of the Sepsityper^®^. Using our new protocol, 81 (95.3%) isolates were identified with a score > 2.0, and 85 (100%) isolates were identified with a score > 1.7; these numbers were 46 (54.1%) and 69 (81.2%), respectively, for the Sepsityper^®^. These results are preliminary, but considering that this novel protocol provides notably high species-level ID rates for Gram-positive isolates, it deserves assessment in a larger-scale study with a variety of platforms for MS-based identification of microorganisms. We are now in the process of making the CMFT protocol more user-friendly and eventually, commercially available.

### II Short-term incubation methods (microcolonies)

Major disadvantages of various protocols for bacterial purification described above are additional hands-on processing time and costs. As an alternative, short-incubation method to identify pathogens from positive culture bottles after very short-term precultivation on solid medium has been proposed [54]. This approach is an excellent compromise between the labor-intensive and costly process of purification of bacterial pellets and the conventional direct smear method after long-term (18 h) subcultures.

According to Idelevich et al. [54], short-term agar cultures with incubation times < 2, < 4, < 6, < 8, and < 12 h resulted in species-level ID in 1.2%, 18.6%, 64.0%, 96.5%, and 98.8% of Gram-positive cocci, and 76.2%, 95.2%, 97.6%, 97.6%, and 97.6% of Gram-negative rods, respectively. Also, after a 5-h subculture on solid medium, correct ID was made in 81.1% of monomicrobial blood cultures, with failure observed with anaerobes and yeasts [55]. More recently, same-day ID and antimicrobial susceptibility testing of bacteria in positive blood culture broths using short-term incubation on solid medium with the MicroFlex LT, Vitek-MS, and Vitek2 systems was reported [56].

This short-term incubation method is reportedly useful for ID of yeast, especially when chromogenic Candida medium was used [57].

## ID of polymicrobial cultures with MALDI-TOF MS

Polymicrobial blood stream infection is not uncommon. Having more than one microorganism in a blood culture bottle delays the ID of all microorganisms, which in turn leads to a worse outcome for the patient [58].

MALDI-TOF MS-based testing of polymicrobial blood specimens that yield a mixed bacterial fingerprint is still challenging. The MALDI Biotyper correctly identified one of the organisms present in nine of 14 polymicrobial cultures [31]. Using the Vitek MS system, one organism was correctly identified in 13 of 28 polymicrobial bottles [38]. More recently, the performances of the Bruker ^®^MBT Sepsityper IVD module for the direct ID of polymicrobial blood cultures were evaluated in 143 polymicrobial cases; 49 cases (34.3%) were completely identified with the module [59].

The short incubation method has been used for polymicrobial cases as well. Although there is a report indicating that both microorganisms present in 43 (86%) of 50 polymicrobial blood culture bottles were correctly identified after short-term culture [60], it is a general view that short incubation method is not successfully applicable to polymicrobial blood stream infections [19].

Identifying the organisms present in polymicrobial blood cultures directly with MALDI-TOF MS is not satisfactory at the moment, which underscores the importance of conventional Gram stains and subcultures.

## The improvements MALDI-TOF MS ID brings to clinical laboratories and patients

The effects of MALDI-TOF MS ID of pathogens in positive blood cultures on laboratory costs and patient outcomes are summarized in a recent review [61]. These effects can be evaluated based on the following measures: 1. time to ID, 2. length of hospital stay, 3. mortality, and 4. financial benefits. In an early report using the Bruker MALDI Biotyper with the Sepsityper kit, the mean reduction in turnaround time to ID was 34.3 h in the ideal situation in which MALDI-TOF was used for all blood cultures and 26.5 h in a more practical setting in which conventional identification from subcultures was required for isolates that could not be directly identified with MALDI-TOF MS [62]. This improvement in turnaround time was shown with a short-incubation method as well [63].

The role of antimicrobial stewardship (AMS) is increasing in human health care; AMS is defined as coordinated intervention designed to improve the appropriate use of antimicrobial agents through promotion of optimal agent selection, dosing, duration and route of administration. Combining rapid ID using MALDI-TOF MS and susceptibility tests with AMS significantly reduced the time to optimal therapy, resulting in a decrease in the mean length of the hospital stay from 11.9 to 9.3 days [64]. Although most studies did not separate the contribution of MALDI-TOF MS and AMS, Beganovic et al. evaluated the impact of MALDI-TOF MS alone versus MALDI-TOF combined with AMS; they found that MALDI-TOF MS plus AMS intervention significantly reduced the overall time to optimal therapy (75.17 vs. 43.06 h) and the overall hospital stay length (15.03 vs. 9.02 days) [65]. More recently, the importance of AMS to enhance the clinical impacts of MALDI-TOF MS was shown with a pre-post quasi experimental study in patients with bacteremia and fungemia [66]. Although the use of MALDI-TOF MS reduced the time to pathogen ID, the use of MALDI-TOF MS alone in a setting lacking an AMS did not afford overall clinical benefits in terms of the time to microbiological clearance and mortality. The clinical impact of MALDI-TOF MS combined with ASM interventions in patients with blood stream infection was demonstrated in a Japanese tertiary hospital as well [67].

Cost savings were definitely realized by implementation of MALDI-TOF MS. Identification costs for a total of 21,930 isolates were directly compared between MALDI-TOF MS and conventional methodologies, considering technologist time in addition to reagent expenses and maintenance costs. MALDI-TOF MS implementation resulted in laboratory savings of $73,646, or 51.7% annually by adopting the new technology, indicating the initial cost of the instrument would be offset in about 3 years [68].

The importance of combining AMS and molecular rapid diagnostic tests including MALDI-TOF MS has been underscored to achieve cost-effectiveness of rapid testing as well [69]. Integrating rapid pathogen ID with MALDI-TOF MS and AMS significantly decreased the hospital cost for patients with Gram-negative bacteremia; $45,709 in the preintervention group vs. $26,162 in the intervention group [64]. In terms of mortality, reports have indicated a significant improvement in bacteremia patients after implementation of combined MALDI-TOF MS and AMS [64, 70]. Of note, a quite recent report showed that mortality improved in Gram-negative bacteremia, but not in Gram-positive cases [71].

## Beyond ID: Increasing roles of LC–MS/MS

Although MALDI-TOF MS is now an essential tool for rapid bacterial ID of pathogens that cause blood stream infection, another important task of microbiology laboratories is antimicrobial susceptibility testing of the causative agents. Although increasing numbers of reports have described applications of MALDI-TOF MS for detection of bacterial resistance to antibiotics [72–78], its contribution is still limited. Three main approaches are used, including detection of antibiotic modifications due to the enzymatic activity of bacteria, detection of antimicrobial resistance based on the specific mass peaks or analysis of mass peak profiles, and detection of resistance with semi-quantification of bacterial growth in the presence of a given antibiotic [72]. Assessment of antimicrobial resistance with MALDI-TOF MS is beyond the scope of this review and has been reviewed elsewhere [72–78].

Although MALDI-TOF MS is more user-friendly than LC/MS/MS, another main MS technology, MALDI-TOF MS has several disadvantages compared with LC/MS/MS. Specifically, MALDI-TOF MS has limited resolving power, and therefore does not necessarily provide sequence-based ID; microbial ID using MALDI-TOF MS is based on spectral fingerprint patterns rather than the identity of each spectral peak. On the other hand, LC/MS/MS is able to identify as many as several thousand peptide sequences, leading to bacterial ID using an appropriate database as reported by Tracz et al. [79].

**Fig. 2:**
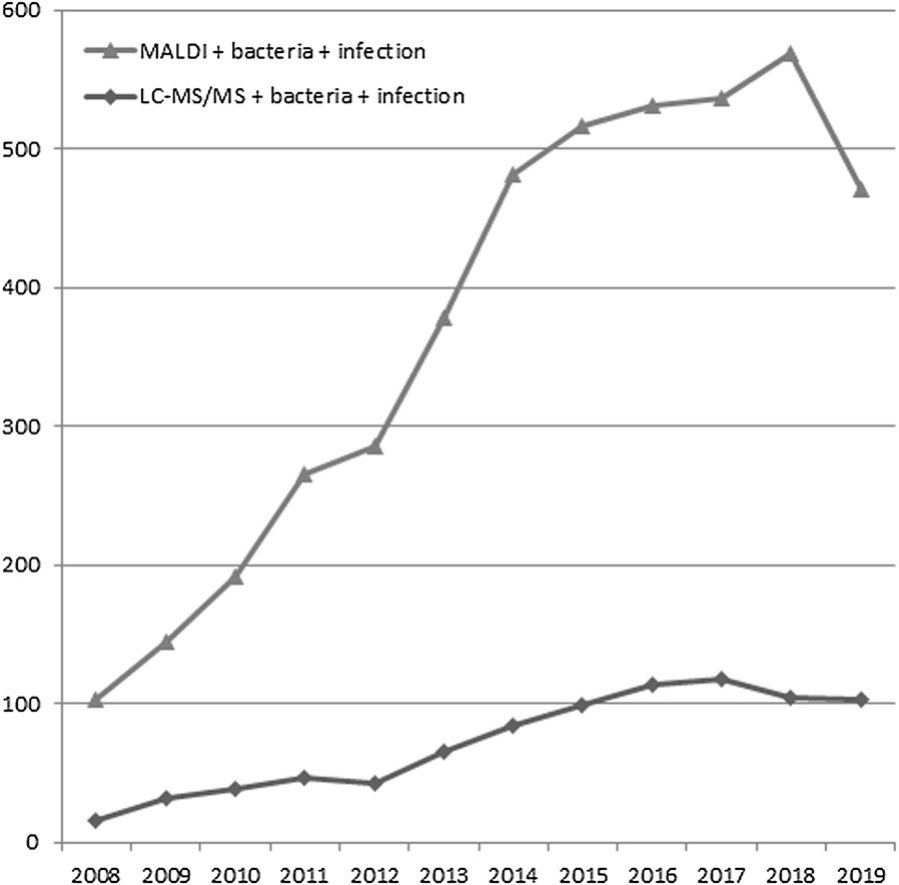
Numbers of publications including three key words, MALDI + bacteria + infection or LC–MS/MS + bacteria + infection identified with the PubMed search conducted on March 25, 2020

Indeed, applications of LC/MS/MS to the microbiology field are increasing according to a PubMed search as shown in Fig. 2. The representative examples of LC/MS/MS protocols used in the clinical microbiology field are shown in Table 2.

**Table 2:** The representative examples of LC/MS/MS protocols used in the clinical microbiology field

Instrument type	Analytical method		Purpose	References
QqQ	SRM/MRM	(Quantitative analysis)	Target analysis (Resistance, Virulence)	[82, 83, 85]
Q-TOF	DDA/DIA	(Qualitative and Quantitative analysis)	Identification, Target analysis	[80–82, 84, 86]
Q or IT-Orbitrap	DDA/DIA	(Qualitative and Quantitative analysis)	Identification	[79, 85]
Q-IT-Orbitrap	DDA/DIA	(Qualitative and Quantitative analysis)	Identification	[84]

*Q* quadrupole, *IT* ion trap, *SRM* Selected Reaction Monitoring, *MRM* Multiple Reaction Monitoring, *DDA* Data dependent acquisition, *DIA* Data independent acquisition

LC–MS/MS has been applied for ID of microorganisms in positive blood culture bottles, with extremely high ID rates [80], and attempts to make this method faster are in progress [81]. Also, LC/MS/MS has advantages over MALDI-TOF MS in terms of detection of microorganism proteins associated with antibiotic resistance. Charretier et al. described label-free selected reaction monitoring (SRM)-based MS to quantify proteins involved in antibiotic resistance mechanisms of *Pseudomonas aeruginosa* clinical isolates [82]. The same group developed an MS method in SRM mode for in-depth characterization of *Staphylococcus aureus* by performing microbial ID, antibiotic resistance detection, virulence assessment, and even epidemiological typing information [83].

Dekker and co-workers have demonstrated that rapid detection of the *Klebsiella pneumoniae* carbapenemase and the colistin resistance protein is possible with LC/MS/MS, and suggested that peptide and/or protein discovery and detection methods should be broadly applicable to allow direct and rapid detection of other resistance determinants [84, 85]. Theoretically, any antimicrobial resistance mechanism involving particular proteins can be detected and quantified with LC/MS/MS using the SRM mode. Indeed, the correlation of detection of beta-lactamase proteins with LC/MS/MS is even better than that with PCR detection of the corresponding genes [86]. Roles for MS-based (or protein-based) and genome-based methods for testing antimicrobial susceptibility and resistance remain to be investigated.

We believe that in addition to MALDI-TOF MS, the roles of LC/MS/MS in the clinical microbiology field will increase. LC/MS/MS, however, is technically demanding and requires investment of high-end instrumentation. Therefore, a substantial amount of education and training is required to take advantage of sophisticated LC/MS/MS in practical clinical microbiology.

To promote the significant benefits that LC–MS platforms bring to a wide range of laboratory medicine and patient care, we initiated a medical mass spectrometrist certification program in the Japanese Society for Biomedical Mass Spectrometry in 2013 with the following objectives: (1) to convey the message that MS-based clinical applications bring significant benefits to laboratory medicine and patient care, and (2) to educate new potential users on the fundamentals of MS-based clinical tests. With a modest amount of additional education and hands-on training, routine use of MS technology can be incorporated into virtually any medical laboratory. As of December 2019, 308 persons from various medical specialties including clinical microbiologists have been certified [87].

## Conclusions

MALDI-TOF MS is now an essential tool for rapid bacterial ID of pathogens causing monomicrobial blood stream infection. Its contribution to detection of susceptibility and resistance to antibiotics, however, is still limited. For this purpose, LC coupled with MS/MS should be more useful and will play significant roles in clinical microbiology in the future.

## Data Availability

Not applicable.

## Abbreviations

AMS: Antimicrobial stewardship
CMFT: Centrifugation and membrane filtration technique
ID: Identification
LC/MS/MS: Liquid chromatography coupled with tandem mass spectrometry
MALDI-TOF MS: Matrix-assisted laser desorption ionization–time of flight mass spectrometry
MS: Mass spectrometry
SRM: Selected reaction monitoring
